# Engineering 3D-Printed Advanced Healthcare Materials for Periprosthetic Joint Infections

**DOI:** 10.3390/antibiotics12081229

**Published:** 2023-07-25

**Authors:** Iván Yuste, Francis C. Luciano, Brayan J. Anaya, Pablo Sanz-Ruiz, Almudena Ribed-Sánchez, Elena González-Burgos, Dolores R. Serrano

**Affiliations:** 1 Pharmaceutics and Food Technology Department, Faculty of Pharmacy, Universidad Complutense de Madrid (UCM), 28040 Madrid, Spain; iyuste@ucm.es (I.Y.); fluciano@ucm.es (F.C.L.); branaya@ucm.es (B.J.A.); drserran@ucm.es (D.R.S.); 2 Orthopaedic and Trauma Department, Hospital General Universitario Gregorio Marañón, 28029 Madrid, Spain; pasanz03@ucm.es; 3 Department of Surgery, Faculty of Medicine, Universidad Complutense de Madrid (UCM), 28040 Madrid, Spain; 4 Hospital Pharmacy Unit, Hospital General Universitario Gregorio Marañón, 28029 Madrid, Spain; almudena.ribed@salud.madrid.org; 5 Department of Pharmacology, Pharmacognosy and Botany, Faculty of Pharmacy, Universidad Complutense de Madrid (UCM), 28040 Madrid, Spain; 6 Instituto Universitario de Farmacia Industrial, Faculty of Pharmacy, Universidad Complutense de Madrid (UCM), 28040 Madrid, Spain

**Keywords:** 3D printing, implant, PJIs, infection, prosthesis, parenteral drug delivery, antimicrobial, coating

## Abstract

The use of additive manufacturing or 3D printing in biomedicine has experienced fast growth in the last few years, becoming a promising tool in pharmaceutical development and manufacturing, especially in parenteral formulations and implantable drug delivery systems (IDDSs). Periprosthetic joint infections (PJIs) are a common complication in arthroplasties, with a prevalence of over 4%. There is still no treatment that fully covers the need for preventing and treating biofilm formation. However, 3D printing plays a major role in the development of novel therapies for PJIs. This review will provide a deep understanding of the different approaches based on 3D-printing techniques for the current management and prophylaxis of PJIs. The two main strategies are focused on IDDSs that are loaded or coated with antimicrobials, commonly in combination with bone regeneration agents and 3D-printed orthopedic implants with modified surfaces and antimicrobial properties. The wide variety of printing methods and materials have allowed for the manufacture of IDDSs that are perfectly adjusted to patients’ physiognomy, with different drug release profiles, geometries, and inner and outer architectures, and are fully individualized, targeting specific pathogens. Although these novel treatments are demonstrating promising results, in vivo studies and clinical trials are required for their translation from the bench to the market.

## 1. Introduction

In the last decade, 3D-printing technologies have been having a great impact on pharmaceutical science and tissue engineering. Three-dimensional printing enables novel manufacturing methods for medicines and advanced drug delivery systems that cannot be fabricated using conventional techniques [1,2,3]. A revolutionizing aspect of applying this technology to this field is the possibility of tailoring personalized medicines that are able to adjust the patient’s treatment according to their phenotypes, genotypes, or lifestyles. Three-dimensional printing reduces equipment costs in comparison with traditional manufacturing methods, which, combined with its versatility, has enhanced the interest in its implementation in medical science, and, subsequently, clinical practice [3]. In the last ten years, there has been a significant increment in the number of publications in this field, from just 13 articles published in PUBMED to more than 1500 [4]. Amongst the different types of drug delivery system, 3D-printed parenteral implants and prostheses have experienced faster development due to the high personalization degree achieved using this disruptive technology [1,2,5,6,7].

The United States Pharmacopeia defines parenteral drug delivery systems as “those preparations intended for injection through the skin or other external boundary tissue, rather than through the gastrointestinal tract so that the drug formulations are administered directly into a blood vessel, organ, tissue, or lesion” [8]. The different routes for parenteral drug delivery administration are: (i) intravenous (IV), (ii) intramuscular (IM), (iii) intradermal (ID), (iv) subcutaneous (SC), (v) intrathecal, (vi) epidural, (vii) intra-arterial, (viii) intracardiac, (ix) intraocular, and (x) intraperitoneal. Those most commonly utilized in clinical practice are SC, IM, and IV, with the latter requiring a higher degree of expertise [9,10,11,12,13,14]. Conventional parenteral formulations rely on the use of liquid solutions and suspensions. However, innovative formulations consisting of liposomes, micelles, and micro- and nanoparticles, along with locally applied implants, are revolutionizing the current therapies, especially in cancer and infectious diseases [12,15]. These novel therapies allow us to overcome the challenges that persist with conventional formulations, for example, the enhancement of drug targeting, adverse effect minimization, and better patient compliance [9,11,14]. However, several hurdles should be kept in mind to meet the clinical requirements for parenteral administration using these novel therapies, such as sterility and isotonicity, combined with the fine control of particle size, high drug loading, physicochemical stability, and scale-up [9,11,12].

Amongst all the innovative formulations that have reached the market for parenteral administration, locally applied implants have demonstrated some of the greatest growth. The global orthopedic implant market size, including reconstructive joint replacements, spinal implants, dental implants, and trauma ortho biologics, was valued at USD 47.8 billion in 2021, which is predicted to almost double by 2030, with a compound annual growth rate of 4.67% [16].

Despite prosthesis implantation being a routine procedure, and the aseptic conditions of the surgical theater, this type of surgery carries a risk of infection, known as periprosthetic joint infection (PJI), which can be caused by the implant itself or the surgical procedure. Bacteria from the patient’s skin can cause PJIs even if the skin is treated with topical antiseptics before the operation, as their efficacy against bacteria in the deeper layers of the stratum corneum is limited [17]. PJIs have a negative impact on healthcare systems, and also on patients’ quality of life, as they must remain hospitalized for long periods, go through new surgeries, and have a higher risk of new comorbidities. The prevalence of PJIs in developed countries is currently around 2% in primary arthroplasties and increases to 4% in revision arthroplasties [18,19,20,21,22]. Infections affecting orthopedic implants, especially during hip and knee replacements, have the worst prognosis, despite hygienic protocols and intraoperative antibiotic prophylactics.

The number of hip replacements has increased rapidly since the year 2000 in most OECD (Organization for Economic Co-operation and Development) countries. On average, the rate of hip replacement increased by 30% between the years 2000 and 2015 (166 operations per 100,000 people), with Switzerland, Germany, Austria, and Belgium having the highest rates for hip replacement (>250 surgeries/100,000 people) [23]. In Spain, the incidence of hip fractures is estimated to be 40,000–45,000 per year, with an annual cost of EUR 1591 million and a loss of quality-adjusted years of life of 7218. A similar incidence is estimated for knee replacement surgery, and is expected to increase soon, especially among populations over 80 years of age [24]. PJIs following hip and knee replacements are major complications for patients, operating surgeons, and healthcare systems, with a substantial economic impact [25]. Although its incidence over the years has dwindled due to modern theater facilities, aseptic measures, and the use of antibiotic-loaded cement, currently, its prevalence varies but can reach up to 30% of cases in developing countries [26,27,28,29]. The overall cost of hip replacement surgery without infection is about EUR 22,927 (EUR 12,148–43,453) while PJIs can increase up to EUR 79,188 (EUR 39,354–141,359). Depending on the country and the health system, PJIs can increase the cost 2–4-fold [24]. The complexity and extended length of the treatment pathway for PJIs place a significant burden on the healthcare system, resulting in an unmet clinical need to find medical solutions to overcome this rising health problem [22,30].

In this review, the treatment and prophylaxis of periprosthetic joint infections (PJIs) will be discussed, with a focus on 3D-printed personalized implants. The main 3D printing techniques will be covered, including the different materials most commonly employed for the 3D printing of parenteral implants and scaffolds.

## 2. Periprosthetic Joint Infections

A standard PJI definition has still not been established which is fundamental for the diagnosis and the proper treatment of this kind of infection. In 2013, the Musculoskeletal Infection Society (MSIS) submitted an initial definition to the International Consensus on Musculoskeletal Infection (ICM). In 2013, a guideline for the diagnosis of PJIs was also published by the Infectious Diseases Society of America (IDSA). A new definition was evaluated at the ICM in 2018 but only was supported by 68% of the delegates [18,19,20]. More recently, in 2021, the European Bone and Joint Infection Society (EBJIS), in collaboration with the MSIS and the European Society of Clinical Microbiology and Infectious Diseases (ESCMID), established a new definition, based on a three-level approach for continuous diagnosis [20]. All these definitions provide great support for and different approaches to research and clinical practice, and their common feature is the establishment of different levels depending on the time of symptom onset. However, none of them are considered the gold standard PJI definition [18,19,20].

There are three main pathways for the infection of the a prosthesis: (i) the perioperative period, which takes place upon inoculation through the patient’s skin, the surgical material, or the air; (ii) hematogenous spread, which can occur even days after the implantation, as pathogens can travel from different parts of the body to the prosthesis; and (iii) direct contact with an infected surface. PJI’s manifestation may vary according to the microbial virulence, ranging from days to months or even years after surgery. Early PJIs are usually related to *Staphylococcus aureus*, streptococci, and enterococci, while delayed PJIs are caused by less virulent organisms such as coagulase-negative *staphylococci* or *cutibacterium* species [18,19].

**Figure 1:**
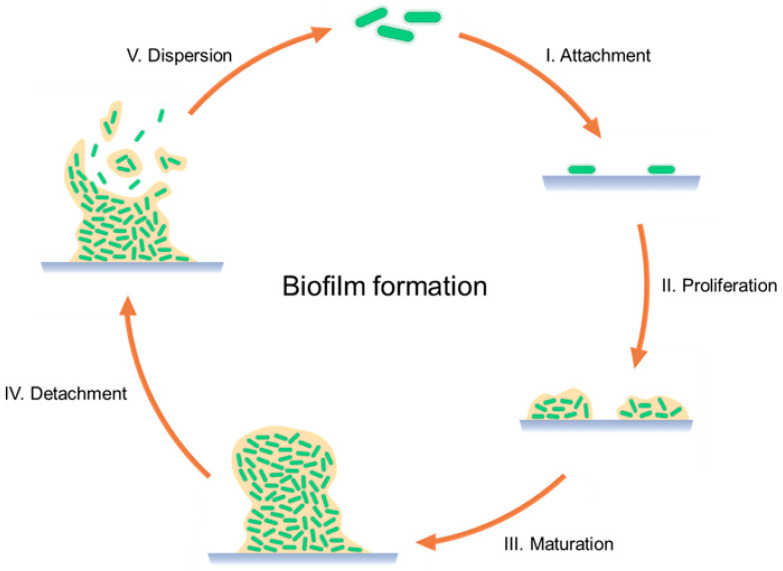
Schematic representation of the biofilm formation process.

The critical mechanism of PJIs is their formation of the biofilm, which consists of 3D clusters of different microorganism species surrounded by an extracellular matrix or polysaccharide glycocalyx, protecting them from the patient’s immune system, antibiotics, and mechanical debridement [18,19,31]. Biofilm formation occurs in four stages: (i) adhesion of the microorganism to the implant surface; (ii) multilayer proliferation and cell-to-cell adhesion, which lead to the formation of microcolonies during the first hours; (iii) maturation of the biofilm into complex communities, which can take place for up to 4 weeks; (iv) cellular detachment happening any time after biofilm’s maturation; and (v) dispersion of the microorganisms to other regions (Figure 1). In mature biofilms, microorganisms present a stationary or non-growing state, due to the accumulation of waste products and metabolic substances. Microorganisms can detach from the biofilm, which leads to the appearance of PJI symptoms, starting with the activation of the immune system and followed by inflammation, edema, pain, and implant loosening [18,19].

Traditional treatments of PJIs consist of a surgical process combined with antimicrobial therapy. For the surgical approach, there are three main strategies: (i) debridement of the infected area with retention of the prosthesis, with a high success rate only occurring when patients meet certain requirements (prosthesis stability, the pathogen’s susceptibility to antimicrobials, the absence of sinus tract or damaged soft tissue, and less than a 3-week symptom duration); (ii) one-stage implant replacement or revision, most commonly used in Europe, in which a single operation takes place to exchange the old prosthesis with a new one; and (iii) two-stage implant replacement or revision, which is considered the gold standard treatment, characterized by implanting a polymethyl methacrylate cement spacer that is loaded with antimicrobials in the joint for several weeks before the insertion of the new prosthesis [18,19,31,32,33,34]. In addition to the surgical process, patients require an antimicrobial treatment that is usually recommended for 12 weeks. The administration route can be intravenous, oral, or via direct irrigation of the infected area [18,19].

Unfortunately, in the two-stage revision, there is only optimal drug release for 24–48 h after insertion [22,23], and it has recently been associated with postoperative renal injury [24,25]. Additionally, biofilm formation on the cement surface is observed [26,27,28]. Cement spacers are temporary and developed with less structural stability, to facilitate later removal, and due to their mechanical properties, there is a high rate of complications, like joint dislocation, spacer fracture, and fracture of the surrounding bone [29].

Traditional treatments do not always present high success rates, due to several factors such as surgical and pathogen variability. Success rates go from 16% to 83% for debridement, from 85% to 90% for the one-stage revision, and from 66% to 95% for the two-stage revision. Moreover, morbidity and mortality risks associated with undergoing repeated surgeries exist [19,32]. For the gold standard two-stage revision, the mortality rate is usually around 4%, although some studies have determined that it can surpass 20% after 5 years and even reach 40% [33]. The main reason why these treatments fail is their inability to identify the pathogens responsible for the infection and to achieve their complete eradication. Moreover, bone cement does not release high enough antimicrobial quantities, allowing for bacterial biofilm growth on the surface of the implant and leading to different complications such as joint dislocation, renal injury, and fracture of the surrounding bone and the spacer itself [18,31].

New therapies are under development and some of them are showing promising results, such as the combination of different antimicrobials, the use of nanoparticles for drug delivery, antimicrobial peptides, and the use of phage and photodynamic therapies. However, no significant improvement in the treatment success rates of PJIs has been achieved recently, as most of the new alternatives still need optimization before reaching clinical practice [31,32]. Hence, there is a clinical need for innovative approaches to prevent and treat PJIs.

## 3. Parenteral Locally Applied Implants

Parenteral implants, also known as implantable drug delivery systems (IDDSs), are medical devices manufactured to replace a missing biological structure, support a damaged biological structure, or enhance an existing biological structure. Parenteral implants can have different or combined functions such as therapeutics, diagnostic, or maintaining mechanical body integrity [9,14,35,36]. It may be difficult to classify them, as some exceptions and combinations exist that can fit into different categories. Considering the time of formation, IDDSs can be divided into the following categories:

- Preformed implants, also known as solid implants. They must be placed through a surgical procedure and can be biodegradable or non-biodegradable. The latter requires another surgery for removal. Their main advantage is their capacity for long-term and sustained-release drug delivery. Release time can be controlled by the material and the drug-loading technique utilized, such as coating or encapsulation [14,15,35].
- In situ-forming implants consist of liquids or semisolids in which the drug is dispersed or dissolved. After SC or IM implantation through a needle, it turns into a solid reservoir at the injection site. Compared to preformed implants, these IDDSs are easier to manufacture and administer, being less painful for patients. In situ-forming implants can be divided into three different groups: in situ cross-linked polymer systems, in situ polymer precipitation, and thermally induced gelling systems [14,15,35].

The materials for developing parenteral implants are a crucial parameter as they should be biocompatible and ideally biodegradable, and the implant should be reabsorbed when the body’s function has been restored so there is no need for removal [9,14]. A range of polymers have been developed for this purpose [13], including poly-DL-lactic acid (PLA), poly-DL-lactic acid-co-glycolic acid (PLGA), and polyethylene glycols (PEGs) the most commonly used [9,13].

IDDSs can be also classified according to the type of drug release mechanism:

(i) Controlled drug delivery via an activation process. In this type of implant, the drug remains in the implant until a physical mechanism (known as external stimuli) induces drug release, such as magnetic fields, ultrasounds, electric fields, temperature, photoactivation, or pressure [9,14,35,37,38].
(ii) Controlled drug delivery via a diffusion process. In this case, the drug diffuses from the core of the implant towards the medium in which it is implanted. The drug release is not easily regulated or modified after implantation as this is a passive diffusion process. For this reason, it is key to evaluate the initial parameters such as the material chosen, the implant shape and geometry, and the drug formulation; all of these in combination will dictate the final drug release kinetics [9,14,35,37].
(iii) Controlled drug delivery via a feedback-regulated process such as bioerosion or pH, in which the drug release is activated by a triggering agent that can be regulated by its concentration [9,14,35].

## 4. Three-Dimensional Printing Technologies

**Figure 2:**
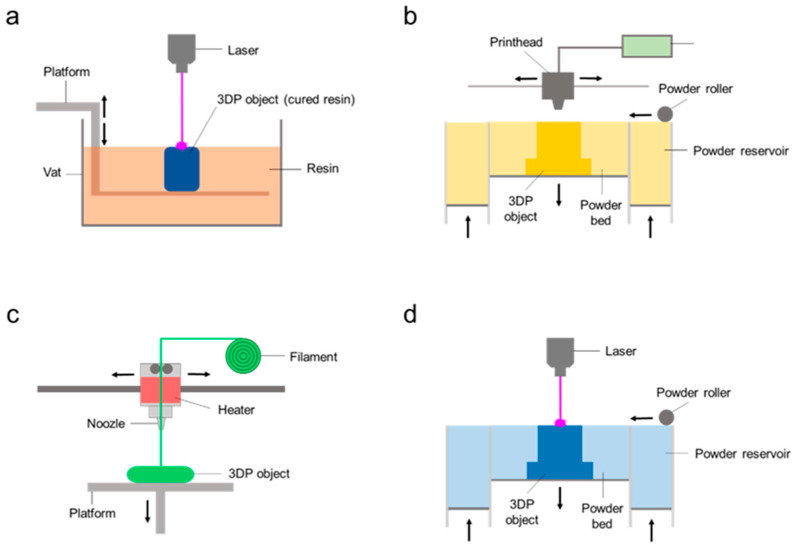
Schematic representation of the main 3D printing techniques for IDDSs: vat polymerization (**a**), binder jetting (**b**), material extrusion (**c**), powder bed fusion (**d**). Key: 3DP, 3D-printed.

There are seven main types of printing technique: (i) vat photopolymerization; (ii) material jetting; (ii) binder jetting; (iv) material extrusion; (v) powder bed fusion; (vi) sheet lamination; and (vii) direct energy deposition. Each of them also comprises different printing systems [39]. Based on the mechanical properties of the joints, direct metal laser sintering (powder bed fusion technique) can be used to print the bone prosthesis itself, replacing conventional implants to manufacture personalized bone prostheses adapted specifically to patients’ physiognomy. However, this type of printing is out of the scope of this review. The most suitable printing techniques for IDDSs are vat photopolymerization, in which a liquid resin is solidified by UV light; binder jetting; material extrusion, like fuse deposition modeling (FDM) and pressure-assisted microsyringes (PAM), also known as semisolid extrusion (SSE); and powder bed fusion, enabling the possibility to fabricate 3D-printed drug-loaded implants (Figure 2) [6,40,41,42].

### 4.1. Vat Photopolymerization

Vat photopolymerization is gaining more interest in the field of pharmaceutics due to its high speed and resolution [43]. The most valuable application of this technology is the development of personalized drug-delivery devices for organ-targeted treatments. Moreover, some progress has occurred regarding other tunable and personalized devices such as microneedles, molds, oral dosage forms, and dental applications. However, the major issue in the manufacturing of parenteral implants and vat photopolymerization is the development of biocompatible materials after long-term contact with the human body [44]. 

This technique includes different 3D printing technologies that share common features, such as the creation of solid objects via light irradiation in a vat of liquid resin. In this process, light triggers the polymerization reaction through the activation of monomer carbon chains of the liquid resin, forming chains of polymers or cross-linking them, resulting in a solid structure. Once solidified, the resin cannot return to the liquid state and the final object can be printed layer by layer [44,45,46]. The main 3D printing methods that use photopolymerization are stereolithography (SLA), digital light processing (DLP), continuous liquid interface production (CLIP), and two-photon polymerization (2PP).

### 4.2. Binder Jetting

Binder jetting (BJT) is defined as an additive manufacturing system, in which a liquid binder is deposited through an inkjet nozzle head onto a powder bed, forming binder–powder agglomerates. Some BJT printers employ a plaster-based powder and a water-based binder. Another layer of powder is spread onto each printed layer, with counter-rotating rolls. Once the printing has finished, a post-processing method is required, as the printed parts tend to be fragile. The object's excess powder is removed, typically via vacuum suction or compressed air, and collected for reuse in subsequent printings. Moreover, an infiltrate is usually injected into the printed object to strengthen the material and improve its mechanical properties [47,48,49,50]. The whole process is easily scalable, which broadens its applications and may increase the interest of different markets and academia [48,49]. Its benefits include the ability to use any powdered feedstock, a high build rate compared to other 3D printing techniques, and the promise of low-cost manufacturing [49].

### 4.3. Material Extrusion

Material extrusion is the most extended 3D printing technique, not only due to its wide applications but also due to the low price of 3D printers and the printing process [51]. This printing technique consists of a tank or reservoir, where the material is contained and pushed through a nozzle that applies pressure. The pressure must be stable and constant to achieve a continuous flow rate of the material, which is required to ensure that it remains in a semisolid state. This material state can be achieved using a heater or chemical reactions. Once the material is deposited on the printing bed or platform, it hardens to a solid state. After each layer is printed, the platform descends and the next layer is deposited over the previous one [6,51]. The main material extrusion methods applied to dosage forms are the following:

- Fused deposition modeling (FDM), where a heater on the material reservoir melts the material. Its advantages include the potential for low-cost manufacturing, the ability to employ any powdered feedstock, and a high build rate in comparison to other 3D printing processes. However, pellets or powders can also be used for FDM. This 3D printing technique is widely used due to the possibility of creating complex structures, which makes it ideal for complex scaffolds or formulations combining different release profiles and the high quality, speed, and reduced cost of the printing process [52].
- Pressure-assisted microsyringe (PAM) or semi-solid extrusion (SSE), consisting of a syringe extruder for depositing viscous or semi-liquid material. The extrusion is achieved via the action of a pressurized-air or mechanical piston. The key factors in PAM 3D printing are the viscosity, viscoelasticity, and apparent elastic limit of the materials [53].

### 4.4. Powder Bed Fusion

Powder bed fusion (PBF) was one of the first additive manufacturing systems developed. It consists of a powder bed, where the action of one or more thermal sources induces the fusion of the powder particles, obtaining a solid material [54,55,56]. Moreover, the system includes a controller for the melting area and mechanisms for the addition and smoothening of the different powder layers (recoaters, hoppers, counterrotating rollers, wipers, or doctor blades) [55]. As with other AM techniques, once a layer is 3D-printed, the bed descends, and another layer is formed on top of the previous layer. The melting of the material can be partial or total, and the heat energy or thermal source comes from a laser or an electron beam [54,55,56]. 

The printing process is carried out in a closed chamber with a steady supply of inert gas, such as nitrogen or argon, to prevent oxidation. Gas flow is also important for removing the condensate formed after the powder melts [54,55]. Depending on the previous factors, PBF is divided into different 3D printing systems:

- Selective laser sintering (SLS) in which a laser acts as the thermal source that binds the powder particles into a solid material. Most SLS systems use CO_2_ laser beams, due to the reduced cost and enhanced power [56,57].
- Selective laser melting (SLM) in which the laser does not fuse the material but it melts the powder into a homogenous mixture [56,58].
- Direct metal laser sintering (DMLS) in which a metal powder is melted by a laser beam, usually requiring high temperatures [56,59].
- Selective heat sintering (SHS) which uses a heater or heated head instead of a laser for melting the powder material via direct contact [56,60].
- Electron beam melting (EBM) in which an electron beam is used as a heat source for melting the powder. This system offers optimal thermal isolation, a high-vacuum environment and high temperatures, which has the advantage of lower residual stress in comparison to laser-based systems [56,61].

## 5. Materials

The materials for 3D-printed parenteral implants are crucial. Biocompatibility is key when selecting an IDDS material, as the body’s immune response should be minimized. Although non-biodegradable IDDSs are commonly used, alternative biodegradable materials can be selected, so when the body’s function has been restored, the implant is reabsorbed and there is no need for removal, minimizing the patient’s risks and the economic cost derived from a second intervention. Other material features that must be considered are the mechanical properties, which may vary depending on the site of application, as well as the capability of promoting cell adhesion and proliferation, and essential tissue regeneration [9,12,14,62]. In this review, we will focus on 3D printing technologies for the manufacturing of locally applied parenteral implants.

**Figure 3:**
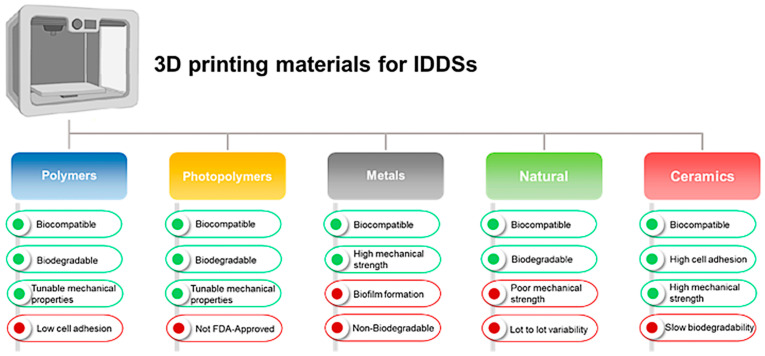
Schematic diagram of the benefits and drawbacks of 3D printing materials for IDDSs.

There are different materials available for the 3D printing of IDDSs, as shown in Figure 3. The benefits and drawbacks will be discussed in the following sections [6,62,63].

### 5.1. Polymers

Polymers are regarded as one of the essential components of IDDSs because of their enhanced pharmacokinetic characteristics. Their main benefit is their capacity for achieving controlled and sustained drug release, and their ability to lengthen or shorten it with ease depending on the polymer’s chemical structure [13,64,65]. Polymers have been used for drug delivery as a reservoir-, diffusion-, and solvent-activated-based drug delivery system, in the form of hydrogels, liposomes, nanoparticles, or implants [66]. The most important polymeric properties that should be considered for parenteral drug delivery are their molecular weight, crystallinity, hydrophobicity, and biodegradability [13].

Not every polymer is suitable for drug delivery implants; only those that are biocompatible, with high physicochemical stability, and that are easy to manufacture, free of pyrogens, and capable of entrapping enough active ingredients are suitable [64]. However, most commercially available polymeric IDDSs for other purposes, such as hormone therapy, are non-biodegradable, requiring a secondary surgical procedure for their removal. This increases the risk of infection and can be detrimental to patients. Additionally, regulatory requirements are more strict for their approval for human use [67]. For this reason, biodegradable IDDSs are becoming a more interesting choice, due to their natural degradation into small molecules that are eliminated easily in the body [64,66].

Polyesters such as polycaprolactone (PCL), poly (lactic acid) (PLA), poly (lactic-co-glycolic acid) (PLGA), and poly (glycolic acid) (PGA) are the most commonly used biodegradable polymers for parenteral drug delivery. The ease of modifying their degradation kinetics based on molecular weight and chemical substitutes enables different drug release profiles, which makes them versatile materials for a wide variety of applications [13,65,66,67]. PLGAs are amorphous polymers in which the residues of lactic and glycolic acid are combined in different ratios, including 50:50, 65:35, 75:25, and 85:15, resulting in degradation times ranging from 3 to 18 months with molecular weights between 7000 and 24,000 Da, resulting in inherent viscosities ranging from 0.09 to 1.7 dL/g [68]. Other biodegradable polymers like polyethylene glycol (PEG) and polyvinyl alcohol (PVA) have also been successfully used in the development of drug delivery systems [66,67,69,70]. PEG is used in combination with other polymers, acting as a plasticizer to reduce the glass transition and printing temperatures [71]. 

Polymers have high printability with a wide range of properties and capabilities. Their use in the 3D printing of biomedical products is greatly extended due to the variety of their properties, and they can be processed using almost every 3D printing technique with low cost and reduced production times [72]. Moreover, polymer blends improve the material’s printability and its properties for drug delivery, such as sustained release [73]. The most common 3D printing technique for polymeric IDDSs is fuse deposition modeling (FDM), where polymers are heated well above their glass transition temperature, becoming malleable and extrudable [74]. Each polymer has different melting temperatures, which vary depending on its molecular weight. These temperatures are summarized in Table 1, as are the different printing properties for each polymer (Table 1) [6,75,76,77]. For FDM, the 3D printing temperature is maintained significantly above the glass transition temperature (Tg), ideally, 20–30 °C, which helps to maintain optimal polymer flow during extrusion [72].

### 5.2. Photopolymers

Photopolymerization is a fast, cheap, and precise manufacturing method, as it takes place at room temperature, and exposure time and area can be easily modified and adapted. The use of photopolymers for biomedical applications such as tissue engineering, cell encapsulation, and drug delivery has gained interest in the last few years [80]. These polymers present modified functional groups, which are required for photopolymerization, as they go through free radical polymerization in the presence of a photoinitiator and light exposure [44,81]. The most common photopolymerizable components are acrylates or methacrylates, located at one or both ends of the monomer or polymer chain [80].

For photopolymerization, biocompatible and biodegradable polymers have been tested, with the most common PEG derivatives including acrylates, acrylamides, and methacrylates such as poly (propylene fumarate-co-ethylene glycol), PEG fumarate, PEG di-methacrylate, PEG urethane-di-methacrylate, and PEG-diacrylamide. PVA, poly (ethylene oxide), and poly (propylene oxide) have also been used for photopolymerization [80]. These modified polymers have enhanced biocompatibility profiles compared to conventional resins, although further studies are required to confirm their safety upon body exposure for prolonged periods [62]. Some of the mentioned components, like acrylamide, are known for their toxicity in monomeric form. To avoid the toxicity of the resins, some alternatives are being used, like diacetone acrylamide (lethal dose (LD_50_) around 3–38 mg/mL) instead of acrylamide (LD_50_ around 0.1–0.26 mg/mL) [82].

Photopolymers are used for 3D printing as liquid resins, composed of monomers, oligomers, and photoinitiators [45,83]. The remaining photoinitiators in the polymer matrix after the photopolymerization process have toxic potential, as they can migrate out of the 3D-printed implant because of their low molecular weight. Novel photoinitiators are under investigation to improve the biocompatibility of photopolymers and their clinical applications. For example, new photoinitiators are being developed based on grafting or condensing low-molecular-weight photoinitiators, achieving linear, dendritic, or hyperbranched polymers [45,84]. Some examples of photopolymers and their photoinitiators are summarized in Table 2.

Optimal printing requires resin viscosity, which is challenging to optimize, as low viscosities are required for a high resolution, but high viscosities are better for a higher mechanical strength of the final product. Due to the lack of resins for biomedical use, the application of photopolymers to biomedicine is still limited [45,83].

### 5.3. Metals

For decades, metals have been widely used for developing and manufacturing orthopedic implants, because of their optimal mechanical properties, plasticity, toughness, corrosion and wear resistance, and biocompatibility [89,90]. Moreover, metallic implants can be combined with drug delivery systems, referred to as IDDS combination products (drugs/devices) by the US Food and Drug Administration (FDA) [91].

Not every metal or alloy is suitable for parenteral applications, as some of them degrade in contact with body fluids, with the consequent release of toxic products. Therefore, high corrosion resistance is a key factor for metal use in IDDSs [90]. The most-used metal for implants, due to their high stability and absence of corrosion, are (i) stainless steel (SS), composed of iron-based alloys with 11–30 wt% chromium and different quantities of nickel, molybdenum, and nitrogen for improving corrosion resistance; (ii) titanium alloys, formed via the addition of interstitial elements (O, N, C, B, and H) and substitutional elements (Al, Cu, Cr, Fe, or Si among others), with improved mechanical properties compared to titanium; and (iii) cobalt alloys, with Cr, Mo, and Ni, with the most common being the cobalt–chromium alloy, with higher corrosion resistance than stainless steel (Table 3) [90,91,92]. However, not every alloy is optimal. For example, in the nickel–titanium alloy, nickel ions are released over time, which are only tolerated in the human body in small quantities [90].

For improving metal properties in parenteral drug delivery, the most common technique is applying coatings to the implant surface, which not only serves as an eluting mechanism for drug delivery but also for increasing biocompatibility, the adhesion of molecules to the surface, osteointegration, and reducing the release of toxic ions [89,90]. For example, metallic implants have been coated with antimicrobials for preventing infections, as microorganisms can grow easily on the surface of these materials [91]. Silver coatings are one of the most commonly used materials, although the presence of silver was found in body fluids and tissues located in the surroundings of the coated implant, leading to different toxic effects [93].

Similar to IDDS use, only some metals can be applied in 3D printing. In comparison with other materials, metals exhibit more difficulties in 3D printing, including a higher cost of the printers and feedstock, irregular surface finishes, challenging qualification and certification, and inefficacy in printing bigger part sizes [94].

For improving their printability, alloys are usually employed, and are also used in combination with other materials (Table 3). The most commonly used alloy is 316 L stainless, due to its higher strength and plasticity, with an elastic modulus and yield strength similar to that of human bone after 3D printing via selective laser sintering (SLS). Titanium suffers an allotropic transformation above 883 °C, changing from α phase to β phase. Alloying elements like Al, C, and O increase this temperature, and Mo, Ta, and Nb decrease it [95]. Modifying this parameter can alter its structure when heated for 3D printing. Ti6Al4V (Ti-64) is the most commonly used titanium alloy, as its mechanical strength increases after heat treatment while maintaining Young’s modulus, which makes it optimal for load-bearing applications. The chromium–cobalt alloy has enhanced mechanical properties after selective laser melting (SLM), compared to using the casting and milling techniques [94,96,97]. New advances are being developed for improving metal 3D printers and printing techniques, which are focused on reducing machine and feedstock costs (which are not expected to decrease soon) and printing speed (such as increasing the energy-beam power or using multiple energy beams), and improving surface finish (developing hybrid machines), qualification, certification, and part size, by employing different approaches, like Ampliforge, a new combined 3D printing and forging technology allowing for the fabrication of a homogenous microstructure without the anisotropy of mechanical prostheses [94,96].

**Table 3:** Three-dimensionally printed metals and alloys for biomedical applications. Key: SLM, selective laser melting; EBM, electron beam melting; SLS, selective laser sintering; 3DP, 3D printing; WAAM, wire arc additive manufacturing; FDM, fused deposition modeling.

Metal	Alloying Elements	Advantages	Fabrication Techniques	Application	Ref
Titanium	Al, Nb, V	Corrosion resistance, high specific strength, low density, microarchitecture, osteointegration	SLM, EBM, SLS	Joint replacement, dental implants, fracture fixation, spinal fusion implants, spinal disc replacements	[91,97,98]
Stainless Steel	Mn, Ni, Ti, Si, Mo, Se, Cr, N	Mechanical strength, non-magnetic, corrosion resistance, fatigue strength	SLM, SLS, binder jetting	Artificial bone, artificial joints, dental implants, fracture fixation, stents, hip stems, spinal implants, cables	[91,97,99]
Iron	Mn, Pd	Ease of manufacturing, mechanical reliability, high fracture strength, high ductility, and high hardness	Binder jetting, extrusion-based 3DP, SLM	Temporary cardiovascular stents and bone tissue engineering	[97]
Magnesium	Al, Zn, Mn, Y, Nd	Biodegradable, mechanical properties similar to human bone and fast degradation	SLM, WAAM, binder jetting, extrusion-based deposition	Orthopedic applications, cardiovascular stents, and bone tissue engineering	[91,97]
Zinc	Mg, Al, Sr	Biodegradable	FDM	Wound closure devices, orthopedic devices, and cardiovascular stents	[97]
Cobalt	Cr, Fe, Ni, Si, Mg, Mo	Mechanical strength, durability, corrosion resistance, fatigue strength, wear resistance	SLM	Joint replacements, stents, pacemaker conductor wires, spinal disc replacements, dental bridgework	[91,97,99]

### 5.4. Natural Materials

Synthetic materials usually face more difficulties for their approval in parenteral drug delivery and tissue engineering, due to the regulatory requirements and the lack of biocompatible and biodegradable options with optimal printability. Natural materials have been broadly studied and applied to drug delivery, as they have a better safety profile in comparison with other materials and are easy to produce and manufacture [100,101].

Natural polymers, composed of polysaccharides, are the most extensive natural material for parenteral drug delivery [100,101,102]. Many of them can be found in our bodies, possessing good biocompatibility and biodegradability profiles with low immunogenicity. Additionally, their properties can be chemically modified and improved when combined with other natural or synthetic materials, which makes them highly versatile materials.

Some of these materials can improve mechanical properties or cell adhesion and differentiation (like hydroxyapatite) or printability (like synthetic polymers (especially for FDM)) [100,101]. Alginate, agarose, hyaluronic acid, chitosan (CS) and its derivatives (carboxymethyl CS, hydroxy butyl CS, catechol CS, or vanillin CS), and cellulose and its derivatives (hydroxypropyl methylcellulose, hydroxypropyl cellulose, ethyl cellulose, carboxymethyl cellulose, and methylcellulose (MC)) are commonly used polymers for drug delivery and 3D printing. Along with polysaccharides, some proteins can be employed in drug delivery systems, such as fibrinogen, silk fibroin, collagen, and gelatin [100,101,102,103].

The use of these materials in 3D printing is attributed to the ease of printing, as they usually do not require high printing temperatures or the use of solvents. This allows for their combination with live cell organisms and, hence, the development of bio-inks for tissue engineering. Commonly, natural polymers in solutions can be printed as hydrogels which are used as scaffolds that enable cell growth. For example, scaffolds of chitosan and hydroxyapatite have been developed for drug delivery and bone regeneration [100,102]. Usually, natural materials are blended with other materials to improve their properties, like sustained drug release. When mixed with synthetic polymers, fuse deposition modeling is used, necessitating high printing temperatures. The printing technique most frequently used when 3D-printed materials are in the form of hydrogels or solutions is pressure-assisted microsyringes (also known as semisolid extrusion) or inkjet printing, which requires low temperatures to prevent water evaporation [100,101,102]. Some examples of the use of natural polymers in 3D-printed parenteral implants are summarized in Table 4.

### 5.5. Ceramics

Ceramics are solid materials formed via the application of heat or heat and pressure and are composed of metal and nonmetal elements. For biomedical applications, ceramics are commonly used, especially those that include calcium phosphates, silica, alumina, zirconia, and titanium dioxide in their composition. This type of material has slow degradability, high cell adhesion, and high mechanical strength, which has led to its use in bone tissue engineering but also for the development of IDDSs [36,111,112].

Bioceramics are ceramic materials applied to biomedicine, and comprise: (i) calcium phosphate ceramics, similar to human bone and can be grouped as hydroxyapatite (HA), beta-tricalcium phosphate (β-TCP), biphasic calcium phosphate (BCP), amorphous calcium phosphate (ACP), carbonated apatite (CA), and calcium-deficient HA (CDHA); (ii) calcium phosphate cement, composed of a solid phase of calcium powder and/or phosphate salts, which forms a paste when mixed with water that is easily manipulated and sets perfectly on bone; and (iii) silica-based glasses consisting of SiO_2_, Na_2_O, CaO, and P_2_O_5_, with optimal properties for bonding and integrating within living bone without resulting in the formation of fibrous tissue and immunoreactivity [111,112,113]. For certain applications, such as scaffold formation, ceramics have some disadvantages, like brittleness, poor degradability, and high tensile strength. To overcome these limitations, ceramic/polymer composites are a commonly used strategy. Certain drugs are also added within 3D-printed structures with ceramics, such as cytostatics or antimicrobials. The addition of biodegradable polymers can modify and improve drug release from ceramic constructs. Some common polymers blended with ceramics are synthetic polymers such as PLA, PCL, PGA, or PLGA; protein-based polymers like collagen or gelatine; and carbohydrate-based polymers like chitosan [111].

Bioceramics have been extensively used for 3D printing. HA, one of the most commonly used materials for scaffold development, is usually combined with polymers to improve their mechanical properties and binding interaction throughout the printing process. Stereolithography is one of the most-used techniques for printing this material, as are extrusion-based methods. However, the melting temperature of HA is over 1000 °C. Thus, HA is mixed with polymers such as collagen, cellulose, PLGA, or PLC, among others, decreasing the printing temperature [113,114,115]. Bioactive glasses, due to their poor mechanical properties and brittleness, are also mixed with other materials for 3D printing, like metals (Zn, Fe), polymers (gelatin, PCL, alginate, polyethyleneimine), or other ceramic materials (β-TCP, HA) [114,115,116]. One of the main problems with the extrusion-based 3D printing of ceramics is nozzle clogging, which can be overcome by increasing the viscosity and homogeneity of the material using higher temperatures and improving material blending [114]. Moreover, the use of ceramics in 3D printing has several limitations related to poor dimensional accuracy and co-printing with living cells, as well as nanoscale control [115]. The main application of ceramics is the manufacture of 3D-printed scaffolds for tissue engineering and drug delivery (Table 5).

## 6. Three-Dimensionally Printed IDDSs for PJIs

**Figure 4:**
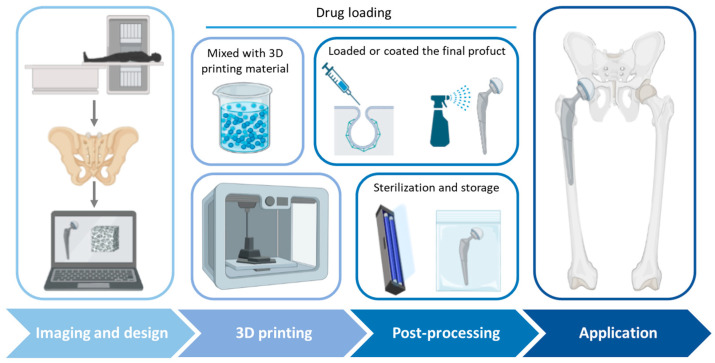
Workflow for 3D printing of IDDSs for PJIs.

Three-dimensional printing has become one of the main technologies in the development of new manufacturing systems and has been extensively applied in medicine and pharmaceutics. One application focuses on the design of drug delivery systems, allowing fast and cost-effective manufacturing and the possibility of creating complex structures and geometries. In recent years, different IDDSs have been developed using 3D printing, like implants, stents, scaffolds, and wound dressings [4,62,121,122]. However, the application of IDDSs and 3D printing to the treatment and prevention of infections, specifically in PJIs, is more recent. In Figure 4, a workflow for the 3D printing of IDDSs for PJIs is illustrated.

The use of IDDSs for the treatment of PJIs has two main approaches:

- The use of 3D-printed scaffolds loaded or coated with antimicrobials, and usually in combination with bone regeneration treatments (Table 6). Inzana et al. [123] compared the antibiotic delivery efficacy of rifampicin- and vancomycin-laden calcium phosphate scaffolds with poly(methyl methacrylate) (PMMA) bone cement, one of the traditional treatments of PIJs. The scaffolds via 3D-printed with binder jetting and showed a higher reduction in pathogenic burden and osteolytic bone resorption. Deng et al. [124] used FDM for the fabrication of polyetheretherketone (PEEK) scaffolds with Ag-modified surfaces. PEEK is a substitute material for bone regeneration, and in combination with Ag nanoparticles, showed optimal osteoblast adhesion and differentiation combined with an antimicrobial effect. Poly-L-lactic acid (PLLA)/ pearl scaffolds, printed using PAM, were mixed with a solution of rifampicin/moxifloxacin-poly lactic-co-glycolic acid (PLGA) microspheres (RM-P) before printing. The scaffolds promoted bone cell adhesion, proliferation, and differentiation and bone defect repair, and showed an anti-infection effect [125]. Zhou et al. [126] developed a PCL scaffold coated with polydopamine (PDA). The coating was used for the adsorption of PLGA microspheres loaded with vancomycin, exhibiting sustained drug release (>4 weeks) with a high antibacterial effect. Moreover, the PCL/PDA scaffold showed higher cell adhesion and proliferation in comparison with plain PCL scaffolds. Topsakal et al. [127] compared the cytocompatibility and the mechanical and antimicrobial properties of four different types of scaffold: (i) polyvinyl alcohol (PVA); (ii) PVA and gold nanoparticles (AuNP); (iii) PVA and ampicillin (AMP); and (iv) PVA/AuNP/AMP. The best outcomes were obtained using PVA/AuNP/AMP scaffolds, resulting in good biocompatibility, osteoinduction, and antimicrobial properties. Liu et al. [128] used tantalum for developing scaffolds, a material already used in arthroplasty. However, this material does not possess antibacterial properties. Porous tantalum scaffolds with chitosan and vancomycin coatings were developed. This combination was shown to prevent bacterial adhesion and biofilm formation. Additionally, the scaffold structure allowed for the generation of a mineralized matrix and osteogenic gene expression. Yang et al. [129] evaluated antibiofilm hydroxypropyl trimethyl ammonium chloride chitosan (HACC)/HA/PLGA scaffolds. The scaffolds showed optimal antimicrobial and osteoconductive in vitro properties resulting in high anti-infection and bone regeneration capabilities in different infected bone defect models. Zhang et al. [130] used PAM for the fabrication of scaffolds with controlled dual-stage release to achieve an antibacterial effect while promoting bone regeneration. β-TCP and PLGA were used as scaffold materials, combined with loaded graphene oxide nanosheets and an osteogenic peptide (p24), which showed an increase in antibacterial sensitivity and osteogenic differentiation.

- The combination of 3D printing with orthopedic implants by creating porous structures or microchannels inside the implants for drug loading, or with different coating methods (Table 7). Hassanin et al. [58] evaluated the optimal conditions of inner reservoirs in drug-delivering Ti-6Al-4V implants via SLM with different internal reservoirs and releasing microchannels (MC). The best hollow implants were those with an MC of 271 μm in diameter, a horizontal surface roughness of 4.4 μm, a vertical surface roughness (Ra) of 9.2 μm, and 1.4% build porosity. Allen [131] developed cobalt–chrome spacers, 3D-printed via SLM, with different antibiotic-eluting reservoir designs. The geometry of the reservoirs affected the API release profile, which could be modulated, resulting in a reduction in the biofilm formation on the spacer surface. Additionally, the spacers had improved mechanical properties in comparison to PMMA spacers. Kim et al. [132] designed a 3D-printed liner for knee arthroplasty. The material used was PLA as the liner material, with different infills in the 3D printing process for creating reservoirs, which were filled with a solution of tetracycline. This liner showed controllable antibiotic release with improved mechanical properties, characterized by higher strength and less brittleness than PMMA, adapted to the patient’s anatomy. To avoid bacterial adhesion in the porous surface of DMLS titanium implants, Guan et al. [133] added antibacterial multilayers to the surface of the 3D-printed implants. This coating consisted of a first phase-transited lysozyme layer and minocycline-loaded multilayers of HA and CS. This IDDS inhibited bacterial adhesion while preserving osteoblast viability and functionality. Griseti et al. [134] compared the bacterial inhibition of 3D-printed porous titanium, tantalum, antibiotic-loaded bone cement, and a smooth titanium alloy. For drug loading, a soaking solution of vancomycin was used in which implants were soaked for one hour. Three-dimensionally printed porous titanium showed higher bacterial inhibition during the first three days in comparison to the other materials. A photopolymer, photocured rigid polyurethane (RPU 60), was used for 3D printing with CLIP spacers with reservoirs for drug release. The channels were loaded with calcium sulfate embedded with gentamicin. This study showed that the reservoir length, diameter, geometry, and quantity modulated the drug release. The longest drug release and antimicrobial effect were achieved with the smallest diameter (0.5 mm), lowest porosity (one channel per side), and greatest length (7 mm) [135]. Instead of directly 3D printing the implant, Maver et al. [136] printed an antimicrobial coating consisting of a hydrogel made of carboxymethyl cellulose, nanofibrillated cellulose, and alginate with clindamycin to be placed on stainless steel and titanium substrates. The 3D-printed coating presented a uniform distribution of clindamycin, with optimal moisture absorption and biodegradability after 7 days. Moreover, no toxicity in osteoblasts was observed along with the antibacterial effects, with an initial burst release combined with a sustained release. Wu et al. [137] 3D printed an antimicrobial hydrogel of chitosan and gelatine on the surface of titanium implants. This coating layer showed an antimicrobial effect against different species of bacteria. Moreover, the hydrogel coating layer allowed for cell adhesion and bone growth, promoting the osteointegration of the prosthesis.

Despite the promising results obtained using the different scaffolds and implants developed for the treatment and prevention of PJIs, all the approaches only target bacterial infections. However, bacteria are the pathogens responsible for 98% of PJIs, while fungi are responsible for the remaining 2%. Despite their low percentage, fungal PJIs are the ones that present a worse prognosis with major complications, being complex in their eradication. Moreover, there is a lack of effective commercialized treatments for fungal PJIs [138,139]. Novel strategies against PJIs should target a combination of antibiotics and antifungals, for those cases requiring broad-spectrum treatment.

Several strategies described in this review were not developed for treating and preventing PJIs but for tissue and bone regeneration. The development of IDDSs combining both antimicrobial effects and the ability to enhance osteointegration would be ideal for the treatment and prophylaxis of PJIs along with faster patient recovery from surgery. Of all the materials described above, synthetic polymers and ceramics have shown the most promising results for IDDS manufacturing in terms of biocompatibility, drug loading capacity, sustained drug release, and mechanical properties. However, further studies are required to obtain systems that mimic closely the physiological properties of the bone microenvironment.

## 7. Future Perspectives

Two-stage revision is considered the gold standard for the treatment of PJIs, with infection eradication rates of over 90%. However, its effectiveness depends on different factors [140]. Despite the advances and the new variety of treatments, disruptive technologies have not been developed that enable the prophylaxis and treatment of PJIs, especially for two-stage revision in acute postoperative infections, where the failure rate is above 50% [141].

3D printing is revolutionizing the pharmaceutical field, with the possibility of changing traditional manufacturing methods. The application of this technology to drug delivery offers different advantages, like tailored drug delivery systems, the development of complex drug release profiles and geometries, with adaptable inner and outer structures, and the lack of prolonged stability profiles [142,143]. Three-dimansional printing is currently being applied and moved forward in the development of IDDSs, even though none of the systems described above have been translated into clinical practice [144,145,146,147].

Despite the promising results of 3D-printed scaffolds and implants for the treatment of PJIs, in vivo studies and clinical trials are required for their approval by regulatory authorities such as the FDA and EMA. Three-dimensional printing offers great versatility for healthcare professionals moving from conventional drug mass-production to customized systems. In the future, it is expected that 3D printing units will be established in hospitals and clinical settings, allowing for in-house 3D printing fabrication and the implementation of parenteral implants and prostheses. Using medical imaging software based on magnetic resonance imaging (MRI) and computed tomography (CT) data and machine learning, the bone and cartilage of knee and hip joints can be segmented and 3D-reconstructed, including advanced features such as cartilage density, volume, and surface [148]. Once the geometry of the desired prosthesis or parenteral implant is established for a specific patient, the printing process step is feasible if suitable inks are developed and provided. At this point, a synergism between large pharmaceutical companies, specialized start-ups, and clinicians is required to develop and target adequate antimicrobial-loaded inks adapted to different healthcare needs to facilitate the clinical translation of 3D-printed precision medicine technology in the management of PJIs.

## Figures and Tables

**Table 1:** Three-dimansionally printed polymer properties for IDDSs. Key: PCL, polycaprolactone; PLA, poly (lactic acid); PLGA, poly (lactic-co-glycolic acid); PGA, poly (glycolic acid); PVA, polyvinyl alcohol; PEG, polyethylene glycol.

Polymer	Printing Temperature	Printability Properties	Biological Properties	Drug Release	Ref
PCL	55–64 °C	During the printing processes, PCL molecules maintain crystal states with low or moderate mechanical properties	Lack of natural peptide motifs that provide specific binding sites for cells	Longer degradation profile than other polymers, suitable for drug release over a year	[6,75,78]
PLA	150–175 °C	High degradation temperature (325–500 °C)	Low cell affinity due to its hydrophobicity	PLA is influenced by the manipulation of its crystallinity degree and mechanical stability	[6,78]
PLGA	>120 °C	The glass transition temperature is reduced with a decrease in lactic acid content in the copolymer	Poor bioactivities (osteoconductive and osteoinductive capabilities)	The time required for the degradation of PLGA is related to the ratio of the monomers used in the starting materials	[75,76]
PGA	220–230 °C	Higher heat distortion temperature than PLA	Improvement in cell adhesion, proliferation, migration, and differentiation for rapid tissue regeneration	The presence of functional moieties in the structural unit allows for tailored degradation rates fitting different applications	[76,79]
PVA	180–228 °C	Suitable for inkjet printing and FDM	Good biodegradability and minimal adverse effects	Suitable for immediate and controlled release	[6,78]
PEG	3–67 °C	Low thermal conductivity	Enhancement of cell encapsulation and it is a widely explored synthetic material for soft tissue repair	Biodegradability and release can be modified by incorporating degradable segments.	[75,77]

**Table 2:** Common photopolymers for drug delivery. Key: PEGDA, polyethylene glycol di-methacrylate polycaprolactone; LAP, lithium phenyl-2,4,6-trimethylbenzoylphosphinate; PI, 2-hydroxy-40-(2-hydroxyethoxy)-2-methylpropiophenone; PEGDAAm, PEG-diacrylamide; pHEMA, Poly(2-hydroxyethyl methacrylate); TPO, diphenyl (2,4,6-trimethylbenzoyl) phosphine oxide; PVA, polyvinyl alcohol; Ru/SPS, tris(2,2′-bipyridyl) dichlororuthenium(II) hexahydrate/ sodium persulfate.

Polymer	Photo-Cross-Linked Moiety	Photoinitiator	Wavelength	Application	Ref
PEGDA	Diacrylate	LAP or PI	365–375 nm	Local anticancer drug delivery and scaffold material	[80,85]
PEGDAAm	Diacrylamide	Irgacure 2959	365 nm	Re-endothelialization-promoting materials and cell encapsulation	[80,86]
Fumarate-co-PEG-co-sebacates	Fumarate	Irgacure 500	365 nm	Controlled drug release systems	[87]
pHEMA	Methacrylate	TPO	370 nm	Controlled drug release systems	[88]
PVA	Methacrylate	Ru/SPS	450 nm	New ink and scaffold material	[80,83]

**Table 4:** Three-dimensional printing techniques and conditions for natural materials. Key: PAM, pressure-assisted microsyringe; FDM, fused deposition modeling; EHD, electrohydrodynamic printing.

Natural Product	3D Printing Technique	Printing Temperature	Printability Properties	Ref
Alginate	PAM	Room temperature	Efficient gelation with a low percentage of material and high-quality mechanical and rheological properties	[100,104]
Chitosan	PAM	Room temperature	Hydrogels with optimal rheological properties, low viscosity, and a fast gelling reaction	[100,101]
	Inkjet			
	FDM (Material blends)	182 °C (with Eudragit), 190 °C (ethyl cellulose), 200 °C (PVA), and 215 °C (PVA)	High thermoplasticity	
Agarose	FDM	55 °C (calcium alginate), 180 °C (PVA)	Low liquefaction temperature	[102,105,106,107]
	PAM	Room temperature or 37 °C		
Cellulose	FDM	190–210 °C (With PCL or PLA)	High crystallinity, elastic modulus, good mechanical properties	[101,108]
	Inkjet	Room temperature		
	EHD			
Hyaluronic acid	FDM	65 °C (PEG and PCL)	Low shape fidelity but unsuitable to produce printable bio-inks	[102,109,110]
	PAM	Room temperature		

**Table 5:** Three-dimensionally printed bioceramic scaffolds. Key: MBG, mesoporous bioactive glass; MFG, metal–organic framework; PLGA, poly (lactic-co-glycolic acid); HA, hydroxyapatite; PAM, pressure-assisted microsyringe; FDM, fused deposition modeling; PCL, polycaprolactone; HPMC, hydroxypropyl methylcellulose.

Blend Composition	Blend Ratio	3D Printing Technique	Printing Temperature	Application	Ref
MGB/MOF	100:0 95:5 90:10 70:30	PAM	Room temperature	Scaffolds with antitubercular drug delivery	[116]
PLGA/HA	9:1	FDM	150 °C	Scaffolds with antibacterial and osteoconductive properties	[117]
PCL/HA/carbon nanotubes	50:45 50:0–5	Nozzle-deposition system	Room temperature	Scaffolds for bone cell growth stimulation	[118]
Ca_3_SiO_5_/HPMC	70:30	PAM	Room temperature	Scaffolds with nano surface structure for bone regeneration	[119]
Ca_7_Si_2_P_2_O_16_/alginate/ pluronic F-127	62:3:35	PAM	Room temperature	Hollow strut-packed bioceramic scaffolds for bone regeneration	[120]

**Table 6:** Three-dimensionallly printed scaffolds for the treatment and prevention of PJIs. Key: PEEK, polyetheretherketone; FDM, fused deposition modeling; PLLA, poly-L-lactic acid; PAM, pressure-assisted microsyringe; PCL, polycaprolactone; PDA, polydopamine; PVA, polyvinyl alcohol; AuNP, gold nanoparticles; AMP, ampicillin; PLGA, poly (lactic-co-glycolic acid); HA, hydroxyapatite; HACC, hydroxypropyl trimethyl ammonium chloride chitosan; β-TCP, β-tricalcium phosphate.

Scaffold Material	Antimicrobial	Drug Loading Technique	3D Printing Technique	3D Printing Conditions	Ref
Calcium phosphate	Rifampin and vancomycin	Mixed in the power before printing or printed onto the scaffold	Binder jetting	Phosphoric acid-based binder solution and bed of calcium phosphate powder	[123]
PEEK	Ag nanoparticles	Coating	FDM	380 °C	[124]
PLLA and pearl	Rifampicin and moxifloxacin	Mixed with a 3D printing material	PAM	150 °C and 110 kPa	[125]
PCL and PDA	Vancomycin	Coating	FDM	Not specified	[126]
PVA	AuNP and/or AMP	Mixed with a 3D printing material	PAM	Room temperature and flow rate of 0.5 mL/h	[127]
Tantalum	Vancomycin	Coating	Not specified	Not specified	[128]
PLGA, HA, and HACC	None	None	PAM	150 °C and 110 kPa	[129]
Β-TCP and PLGA	Chlorhexidine	Mixed with a 3D printing material	PAM	Angle of 90°; printing speed of 10–14 mm/s, and pressure of 1.5 MPa	[130]

**Table 7:** Three-dimensionally printed implants for the treatment and prevention of infections. Key: SLM, selective laser melting; PLA, poly (lactic acid); DMLS, direct metal laser sintering; RPU 60, photocured rigid polyurethane; CLIP, continuous liquid interface production; PAM, pressure-assisted microsyringe.

Implant Material	Antimicrobials	Drug Loading Technique	3D Printing Technique	3D Printing Conditions	Ref
Ti-6Al-4V	-	Reservoirs and micro-channels	SLM	Argon atmosphere, 1075 nm, a constant beam spot size of 70 μm, 200 W, printing speed of up to 4000 mm/s, and layer thickness of 20 μm	[58]
Cobalt–chrome (Co28Cr6Mo)	Gentamicin	Syringe injection in reservoirs	SLM	Not specified	[131]
PLA	Tetracycline	Syringe injection in reservoirs	Not specified	Not specified	[132]
Ti–6Al–4V	Minocycline	Coating	DMLS	Argon atmosphere, 1054 nm, 200 W, laser scanning speed of 7 m/s, and laser spot size of 0.1 mm.	[133]
Titanium	Vancomycin	Soaking solution	Not specified	Not specified	[134]
RPU 60	Gentamicin	Syringe injection in reservoirs	CLIP	Not specified	[135]
Stainless steel and Ti–6Al–4V	Clindamycin	3D-printed coating	PAM	0.25 mm nozzles and room temperature	[136]
Titanium	Chitosan and gelatine	Coating	PAM	50 °C, pressure of 0.3 MPa, and printing speed of 3.3 mm/s	[137]

## Data Availability

Not applicable.
